# A Scoping Review of Factors Affecting COVID-19 Vaccination Uptake and Deployment in Global Healthcare Systems

**DOI:** 10.3390/vaccines12101093

**Published:** 2024-09-25

**Authors:** Chikondi C. Kandulu, Laura J. Sahm, Mohamad M. Saab, Michelle O’Driscoll, Megan McCarthy, Gillian W Shorter, Emma Berry, Anne C. Moore, Aoife Fleming

**Affiliations:** 1 Pharmaceutical Care Research Group, University College Cork, College Rd, T12 K8AF Cork, Ireland; l.sahm@ucc.ie (L.J.S.); michelle.odriscoll@ucc.ie (M.O.); a.fleming@ucc.ie (A.F.); 2 Mercy University Hospital, Grenville Place, T12 WE28 Cork, Ireland; 3 Catherine McAuley School of Nursing and Midwifery, University College Cork, Brookfield Health Sciences Complex, T12 AK54 Cork, Ireland; msaab@ucc.ie (M.M.S.); meganmccarthy@ucc.ie (M.M.); 4 School of Psychology, Queen’s University Belfast, Belfast BT9 5BN, UKe.berry@qub.ac.uk (E.B.); 5 School of Biochemistry and Cell Biology, University College Cork, T12 XF62 Cork, Ireland; anne.moore@ucc.ie; 6 National Institute for Bioprocessing Research and Training, A94 X099 Dublin, Ireland

**Keywords:** COVID-19 vaccines, healthcare systems, vaccination strategies, vaccine rollout, vaccine implementation, vaccine access, vaccine deployment, vaccine campaign, immunization programs, health policy

## Abstract

**Introduction**: COVID-19 vaccines were rapidly developed and deployed on a large scale during a global crisis. A range of deployment strategies were used globally to maximize vaccine uptake. In this scoping review, we identify and analyze the main healthcare system and policy factors that guided and influenced COVID-19 vaccination deployment and uptake globally. **Materials and Methods**: JBI guidelines, Preferred Reporting Items for Systematic Reviews and Meta-Analyses extension for Scoping Reviews (PRISMA-ScR), and the population, concept, and context (PCC) framework were applied. Studies on individual COVID-19 vaccination factors, such as vaccine hesitancy, were excluded. The search was last conducted in May 2024 yielding 26,686 articles from PubMed, Embase, CINAHL, Scopus, and COVID-19 websites. A total of 47 articles and 3 guidance documents were included. The results of the thematic analysis were mapped to the Consolidated Framework for Implementation Research (CFIR). **Results**: The results found the following healthcare system and policy factors as integral to COVID-19 vaccination: types of vaccine products, healthcare workforce capacity, procurement strategies, distribution and cold-chain capacity, partnership, coordination, and leadership, information, communication, and registration strategies, delivery models, organizations, the existing health systems and policies on prioritization of at-risk groups and deployment plans. **Discussion**: Globally, COVID-19 vaccination programs responded to the pandemic by leveraging and reforming the existing healthcare systems, relying on strong leadership and global cooperation (such as the COVID-19 Vaccines Global Access Initiative). Deployment was enabled by effective communication and adoption of innovative technologies using data-driven policies to create high vaccine demand while overcoming limited vaccine supply and rapidly adapting to uncertainties.

## 1. Introduction

The rapid emergence of the SARS-CoV-2 virus required a fast and effective medical response. Vaccines were a crucial part of this response to curtail disease severity and mortality caused by this viral infection and ideally aimed to prevent it [1,2]. COVID-19 vaccines were successfully developed and were first granted Emergency Use Authorization (EUA) in 2020 within 12 months of the pandemic outbreak. As of September 2023, Spikevax® (Moderna Tx, Inc, Cambridge, MA, USA), Nuvaxovid® or Covovax® (Novavax Inc, Gaithersburg, MD, USA), and Comirnaty® (Pfizer Manhattan, New York, NY, USA) and (BioNTech, Mainz, Germany)) received full licenses from the Food and Drug Administration (FDA) for all persons aged ≥6 months [3]. Despite the EUA, there were persistent vaccine supply challenges, especially between 2021 and early 2022, causing substantial delays in deployment in many regions of the world [4]. To address this, novel approaches were key to successful COVID-19 immunization campaigns and programs. 

The COVID-19 Vaccines Global Access Initiative (COVAX) is one of three pillars of the Access to COVID-19 Tools (ACT) Accelerator, launched in April 2020 as part of a global, coordinated pandemic response [5]. COVAX brought together governments, global health organizations, manufacturers, scientists, the private sector, civil society, and philanthropic entities to provide equitable access to COVID-19 diagnostics, treatments, and vaccines [6]. It was co-led by the Coalition for Epidemic Preparedness Innovations (CEPI), the Global Alliance for Vaccines and Immunization (Gavi), and the World Health Organization (WHO), alongside a key delivery partner, the United Nations Children’s Fund (UNICEF) [7]. One of COVAX’s goals was to provide access to nearly two billion COVID-19 vaccine doses by the end of 2021 [6]. COVAX guided countries with continuously updated policies, guidelines, and strategies to ensure increased COVID-19 vaccine uptake and deployment. National Deployment and Vaccination Plans (NDVPs) were formulated to introduce COVID-19 vaccines and design strategies for the deployment, implementation, and monitoring (uptake, scheduling, and boosting) [8]. COVAX aimed to ensure the alignment of the deployment plans with funding and other national COVID-19 recovery response and support plans [8].

Naik et al. identified the COVID-19 vaccine deployment as a continuous process, requiring real-time information and changes in strategy as the planners (i) estimate the need, (ii) secure vaccine supply, (iii) distribute the vaccines, and (iv) implement post-market surveillance [9]. COVID-19 vaccines were procured worldwide to ensure a steady supply despite the scale-up challenges [10,11]. Due to uncertain epidemiologic settings and vaccine supply, a global framework was established to prioritize and allocate these vaccines according to emerging evidence on specific vaccines, and the economic impact of the pandemic [12]. Deployment of the vaccine in mass vaccination centers was widely adopted to decrease and control the spread of the COVID-19 disease [13]. 

Individual countries implemented various deployment strategies and timelines to maximize COVID-19 vaccine uptake with limited resources. For example, the United Kingdom (UK) and Israel responded rapidly and early by leaning on existing healthcare systems and early rollout plans that included the COVID-19 vaccine procurement, data, and a variety of providers [14]. The United States of America (USA) and Brazil downplayed the threat of the pandemic and the importance of vaccines, leading to a delay in vaccine response relative to some early-moving countries [15].

Few studies have investigated and highlighted different system-level factors critical for the successful introduction, deployment, and uptake of vaccines globally. Kochhar et Al. conducted a global review study on the programmatic, regulatory, safety, and ethical considerations of maternal vaccines in 2019 [16]. In 2022, a study developed a road map to support policies and delivery of seasonal influenza vaccines in the East Mediterranean Region (EMR) [17]. In 2023, Bhatt et al. conducted a scoping review identifying guidance for establishing a country’s readiness to deploy different vaccines including the COVID-19 vaccine globally [18]. These studies reported the following healthcare system factors that were key to vaccination deployment and uptake: legal, political, professional, sociocultural, communication, financing, sustainable integrative healthcare, vaccine characteristics and logistics, programmatic planning, program monitoring and evaluation, human resources, and disease burden [16,17,18]. Healthcare systems have a critical role in implementing national and international COVID-19 vaccine deployment, alongside policies and guidelines [19,20]. Much of the literature has focused on individual factors affecting COVID-19 vaccine uptake, and some literature identified factors influencing COVID-19 vaccine deployment. However, most of these were evaluating either small settings (few countries or specific levels of healthcare) and/or specific components of the COVID-19 vaccine implementation process. Without a thorough understanding of healthcare system-level facilitators and barriers, we will struggle to learn from the best vaccine deployment practices during this pandemic. Thus, our findings will provide an overview of the healthcare systems’ issues that need to be considered when deploying vaccines in an immunization campaign in both pandemic and non-pandemic settings.

The specific objectives of this review were to explore the (i) healthcare system factors and (ii) policy factors that influenced COVID-19 vaccination deployment and uptake globally. This enables us to evaluate the characteristics of successful strategies and policies, relating to healthcare systems, that were adopted to maximize vaccination deployment and uptake. This will provide insight for healthcare and policy stakeholders to plan and augment their existing and future vaccine policies and deployment strategies supporting improved delivery and uptake of immunization programs.

## 2. Materials and Methods

This scoping review was conducted according to JBI guidelines for conducting scoping reviews [21] and reported using the Preferred Reporting Items for Systematic Reviews and Meta-Analyses extension for Scoping Reviews (PRISMA-ScR) checklist Supplementary Materials Table S3 [22]. The protocol was registered in the Open Science Framework (OSF) https://doi.org/10.17605/OSF.IO/K28CS in December 2023, accessed on 10 January 2024. 

### 2.1. Eligibility Criteria

The population (or participants), concept, and context (PCC) framework was used. 

Population: Decision-makers/policymakers/managers of COVID-19 vaccine deployment.

Concept: Peer-reviewed articles of any design were included if the information reported on the following elements of the process of COVID-19 vaccination:

- access,
- delivery/deployment,
- distribution/logistics,
- policy/governance,
- implementation/rollout,
- procurement supply chain and cold chain.

Articles focusing on equity, individual uptake, where the reporting period was before the COVID-19 vaccine rollout, and modeling of the COVID-19 vaccine were excluded.

Context: Any country.
Articles focusing only on small regions of a country were excluded.

Protocols, commentaries, perspectives, case studies, abstracts, viewpoints, letters, supplement issues, correspondence, features, short communications/news, essays, forums, editorials, and opinion articles were excluded.

As there is no formal definition of the term vaccine deployment, for the purposes of this study, vaccine ‘deployment’ was defined as the process of defining the type of vaccine to be procured and its cost, transportation, vaccine supply, distribution, and cold-chain management, defining the target population for vaccination and delivery (methods, where and who to deliver) and monitoring and evaluation of the vaccines. Vaccine ‘uptake’ was defined as the practical issues (affordability, availability, ease of access, service quality, and health workers) of ensuring that the vaccines reach the population adequately and efficiently, encompassing communication, registration and appointment booking, and delivery methods.

### 2.2. Study Searching

A systematic search was conducted in PubMed, Embase, CINAHL, and SCOPUS using the National Library of Medicine’s MeSH (Medical Subject Headings) and synonyms of the keywords ‘health system vaccine rollout terms’ and ‘COVID-19 vaccine’ (see Supplementary Materials Table S4). We restricted to articles published between 2020–2024. The complete strategy was peer-reviewed through the Peer Review of Electronic Search Strategies (PRESS) checklist by a qualified medical librarian [23]. Articles were imported into Covidence, (https://www.covidence.org/ accessed on 25 July 2024, Veritas Health Innovation, Health Innovation, Melbourne, Australia, 2024), a web-based collaboration software platform that streamlines the production of systematic and other literature reviews. The map was generated using QGIS (version 3.22) “QGIS.org (2020). QGIS Geographic Information System. Open-Source Geospatial Foundation Project, Switzerland. http://qgis.org” accessed on 31 July 2024. A focused gray literature search was conducted on the websites of the WHO, the Elsevier COVID-19 Healthcare Hub (https://elsevier.health/en-US/covid-19/home) accessed on 31 May 2024, Coronavirus Research Database ProQuest (https://www.proquest.com/coronavirus) accessed on 31 May 2024 and COVID-19 global research and information resources (https://covid19.researcher.life/) accessed on 31 May 2024. Gray literature was defined as publicly available non-peer-reviewed literature. Non-English language articles were translated via Google Translate.

### 2.3. Selection of Sources of Evidence, Data Charting Process and Data Items 

Duplicates were removed in Covidence. The first step included a title and abstract screen that was carried out by two independent reviewers (CCK and another reviewer), with a third reviewer brought in to resolve any discrepancies in Covidence. Any further discrepancies were discussed collectively to reach a consensus. The second step included full-text screening, with two independent reviewers (CCK and another reviewer) again screening all papers. A third reviewer was brought in to resolve any discrepancies in the full-text review through discussion. Data extraction was conducted by CCK on all included studies, and this was cross-checked by members of the team for accuracy. 

A template for data extraction was developed, which included: (i) study identification (ID) Digital Object Identify (DOI), (ii) title, (iii) author details, (iv) article publication year, (v) study setting, (vi) study source, (vii) study period, (viii) funding source, (ix) conflict of interest, and (x) themes of interest (e.g., types of COVID-19 vaccines used, model of vaccine delivery, healthcare workforce who delivered the vaccines, vaccine policies, vaccine procurement strategies, vaccine distribution, and challenges and successes to COVID-19 vaccine rollout). 

Results were synthesized using a thematic approach focusing on the identification of relevant themes related to governance, procurement, delivery models, healthcare workers, types of vaccines, supply chain, policy, prioritization groups, challenges, and successes of COVID-19 vaccine uptake. Through an iterative analytical strategy, drawing on semantic relationships between these factors, the themes were mapped into the five domains (innovation, outer setting, inner setting, individual, and implementation process) of the Consolidated Framework for Implementation Research (CFIR) [24]. The CIFR is a practical framework with a menu of 39 constructs organized into 5 domains that can be used to guide systematic assessment of the setting-level barriers and facilitators that predict anticipated or actual implementation outcomes through the domains of innovation, outer setting, inner setting, individual and implementation process [24]. We used this healthcare system determinants framework to group our results on the determinants of COVID-19 vaccine deployment at the healthcare setting level. 

## 3. Results

**Figure 1:**
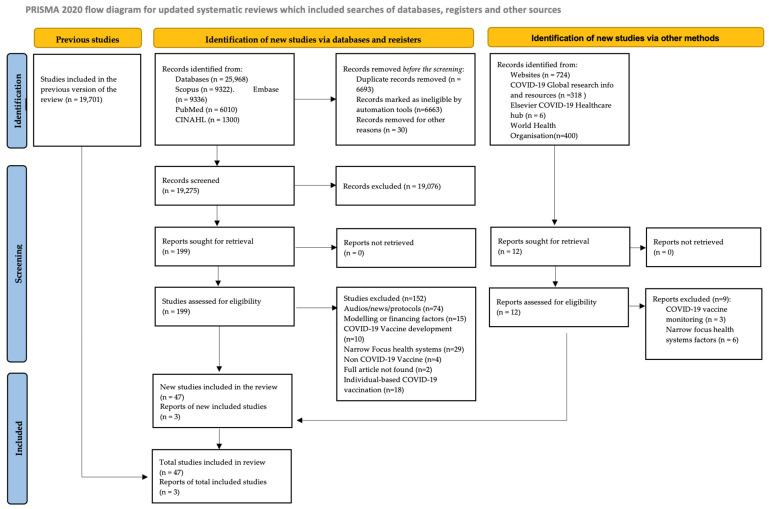
PRISMA diagram describing the article selection for the review of health system factors affecting COVID-19 vaccine deployment and uptake.

A total of 47 articles and 3 guidance documents were included in the final analysis (Figure 1). 

### 3.1. Study Characteristics 

**Figure 2:**
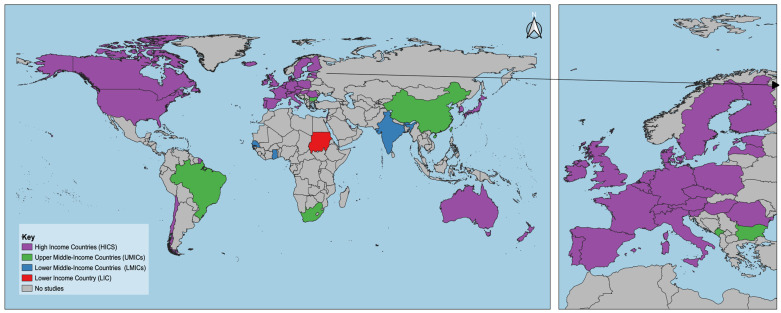
Countries represented in the included records. The arrow shows a zoomed version of European countries, which were mostly included in the study.

The 50 records comprised articles (62%, N = 31), reviews (24%, N = 12), reports (2%, N = 1), policy analyses (6%, N = 3; Supplementary Materials Table S1), and gray literature guidance documents from the WHO website (6%, N = 3; Supplementary Materials Table S2). Of 50 records, 56% (28/50) were at the country level [25,26,27,28,29,30,31,32,33,34,35,36,37,38,39,40,41,42,43,44,45,46,47,48,49,50,51,52], 28% (14/50) had a global focus [8,10,12,20,53,54,55,56,57,58,59,60,61,62], and 16% (8/50) were regional (5 from Europe, 2 from Africa, and 1 from Eastern Mediterranean) [11,63,64,65,66,67,68,69]. A total of 51 countries were included in this study: 40 from high-income countries (HICs), 5 from lower-middle-income countries (LMICs; i.e., Bhutan, Lebanon, India, Ghana, and Senegal), 5 from upper-middle-income countries (UMICs; i.e., South Africa, Montenegro, China, Brazil, and Bulgaria), and 1 from lower-income countries (LICs; i.e., Sudan), as defined by the World Bank Country income classification [70] (Figure 2). Information from the articles ranged from COVID-19 vaccine deployment and delivery, human resource management and implementation, procurement, vaccination infrastructure, immunization information systems, distribution, system leadership, and policies in the period between late 2020 and 2023. A summary table presenting the included studies is presented at the end of the paper (Supplementary Materials Table S1).

### 3.2. Determinants of COVID-19 Vaccine Deployment and Uptake at the Healthcare Level Globally

Facilitators and barriers to COVID-19 vaccine deployment and uptake were systematically categorized using CFIR and grouped into its five domains (Table 1) [24].

(1) **The innovation domain of the CFIR**

*Types of vaccine products*

The strategies that facilitated COVID-19 vaccine deployment during the pandemic included firstly, the use of multiple COVID-19 vaccine products with different platforms including mRNA vaccines Comirnaty^®^ (Pfizer/BioNTech, Cambridge, MA, USA) and Spikevax^®^ (Moderna, Cambridge, MA, USA); recombinant adenovirus vector vaccines Vaxzevria^®^, (AstraZeneca plc, Cambridge, UK), Jcovden^®^ (Janssen Vaccines, Leiden, The Netherlands) and subunit protein vaccines such as Nuvaxovid^®^ (Novavax, Gaithersburg, MD, USA), among others [26,27,28,29,30,31,32,44,48,49,50,51,52,55,61,63,64,66,68,71]. Secondly, the use of single-dose COVID-19 vaccines such as Janssen’s Jcovden^®^ globally facilitated deployment due to ease of logistics and distribution, cost, easy access, and to prevent attrition of vaccinees [58,64,67]. Thirdly, extending the time between doses enabled time before the next vaccine batch was accessible during a period of limited supply, thus facilitating deployment and uptake [58]. Finally, mixed vaccination enabled continuous vaccine supply in the period of limited supply and gave people options to choose and use vaccines with less reactogenicity for those with comorbidities globally [58]. Mixed vaccination was significant in settings with a shortage of vaccines or where there were safety concerns, such as with Vaxzevria^®^ (to allow those who had a first dose to complete their vaccine series with a different vaccine). Mixed vaccination has been associated with higher cellular and humoral immune responses without significantly increasing the adverse reactions [72].

Some of the challenges related to the type of vaccine include uncertainty about the vaccine’s safety and efficacy; for example, Vaxzevria^®^ was suspended in Europe due to safety concerns [31,52,64]. Temporary safety concerns in Europe and the USA for some vaccine types affected vaccine uptake in some African countries such as Malawi and South Africa [64]. Using a variety of vaccines led to challenges with logistics, record keeping, cost of vaccines and infrastructure, attrition of recipients and training of health personnel globally [10,59,67]. This is because different vaccines had different requirements from existing immunization programs and therefore required alternative refrigeration, handling, and delivery approaches impacting vaccine deployment and uptake.

(2) **The individual domain of the CFIR**

*Healthcare workforce capacity*

Countries reorganized their healthcare services including COVID-19 vaccination to maximize the delivery of their essential health services globally in 2021–2022. Strategies such as employing and training both health and non-health workers, retired workers, students, allied health professionals, pharmacists, dentists, midwives, and volunteers (army, data collectors) enhanced COVID-19 vaccine deployment [11,20,42,46,66]. Countries like Ireland [66,68], Germany [20,52,57,66,68,69], France [20,32,66,68,69], Italy [32,57,66,68], the UK [20,66,68], the USA [20,43,57], Chile [29], and Canada [31], were highly effective at using midwives and pharmacists to vaccinate a large population of people. Healthcare workers played a significant role in vaccine uptake as not only did they deliver vaccines and were key communicators with the public, but they were also vaccine recipients, enabling trust in the health system and vaccine confidence, hence influencing uptake. Ghana [48] and Europe [66] invested in vaccination, training, mental health, incentives, and the general welfare of health workers.

The most common challenge to vaccination deployment was the shortage of personnel globally [26,39,61,66]. This was worsened by the large workload to manage COVID-19 cases in hospitals and run day-to-day essential health services including vaccination, constraining the workforce [28,57,66]. Furthermore, the lack of a trained workforce was an issue in India [26,61], Australia [46], and most African countries [61,64], as the new vaccines had special cold chain and handling requirements.

*Partnership, coordination, and leadership*

Strong partnerships with different stakeholders facilitated vaccine uptake in Chile [29], Europe [11,65], Canada [31], France [32], South Korea [40,41], Israel [32,37], Italy [32], Finland [34], Spain [32], and Australia [28]. For example, the Canadian health authority was well coordinated and organized, partnering with different relevant stakeholders including the federal government, which was responsible for funding healthcare, vaccine approval, purchasing, and distributing vaccines to provinces. Canadian provincial governments developed policies to prioritize and distribute the COVID-19 vaccines, the National Advisory Committee on Immunization (NACI) guided vaccines, Transport Canada assisted with vaccine distribution to vaccination sites, the Ontario Ministry of Health developed and refined eligibility criteria, which were implemented by regional vaccine task forces, Public Health Ontario which was providing vaccine information and communication to people, in partnership with Indigenous leaders [67]. 

Coordination of the vaccine deployment program also increased its success. Centralized systems of COVID-19 vaccination implementation facilitated high vaccine deployment and uptake due to unity, efficiency, and coordination at a single level using one strategy. Israel [37] and Bhutan [35] partially attributed their vaccination success to centralization. Other countries that had a centralized system and successful COVID-19 vaccine deployment include Chile [29] and South Korea [40,41]. The following countries had a decentralized system: Canada [31,38], the USA [33], Finland [34], Hungary [69], Romania [69], Sweden [69], Switzerland [47], Austria [47], and Germany [47,69], at either the federal, state, province, regional, or district level. The USA’s challenges in vaccine deployment have been attributed to decentralization, a lack of sharing of vaccine information, lack of coordination and poor communication between state and local health officials, different policies, and competing vaccine procurement and supply at the federal level [33,39]. India [27,36], Italy [32,69], Australia [46], Spain, and France [32,69] had mixed decentralized and centralized systems.

The low political will of leaders and politicization of the COVID-19 vaccine deployment process was a challenge in many countries. In Brazil, President Bolsonaro was reluctant to launch large-scale COVID-19 vaccine campaigns and fuelled conspiracy theories as he denied the existence of COVID-19, causing confusion, disagreements, and hesitancy among the people [30]. In the USA, President Trump contradicted experts in the media about vaccination and was not supportive of COVID-19 vaccines [33]. This negatively impacted uptake, deployment, and policy, and as a leader and policymaker, not only did he deliver fewer vaccine doses than promised, but his stance contributed to reduced vaccine confidence in the USA [73]. Most African leaders allocated limited funds for immunization campaigns and relied on COVAX and external donors [64]. This hindered vaccine deployment efforts as it affected vaccine supply and delivery. The politicization of the COVID-19 vaccine development, manufacturing, regulation, and leadership in many countries globally also hindered vaccine deployment and uptake [30,39,59].

Challenges in creating and maintaining partnerships, public health reforms, problems with healthcare leadership, and vaccine politics in the USA (New York) [33,39], India [26], and other African countries [64] were reported to hinder vaccine uptake. For example, challenges existed with enrolling local healthcare providers as COVID-19 vaccine providers due to concerns around complex storage, handling, and administration requirements in New York [39]. 

A dispute between the president and governors in Brazil affected the stakeholders’ (the health system, pharmaceuticals, and ANVISA [the Brazilian healthcare regulatory body]) coordination and planning of the COVID-19 vaccination campaign, fuelling doubts and distrust in the quality of the vaccines in people impacting both vaccine deployment and uptake [30,51]. Another reported challenge was the inability of many governments to reform and reorganize their health system in the face of the pandemic, such as in India [26]. Many countries, including Austria and Germany, had a fragmented organizational structure during the COVID-19 pandemic, where they moved between central government governance and federal state governance, leading to fragmented vaccine policy implementation and procurement impacting deployment [47].

(3) **The implementation process domain of the CFIR**

*Procurement strategies*

The need for a vaccine led to different vaccine procurement strategies between 2020–2023. Some of the strategies that facilitated vaccine supply and availability included bilateral agreements between manufacturers and individual countries (for example, South Africa [25,56], India [26,36,61], Bhutan [35], Chile [29], and most HICs [11,31,37,46,49,61,63]). Multilateral deals between manufacturers and COVAX (global level) [6,10] and the Pan American Health Organization Revolving Fund (PAHO RF) [61], the African Vaccine Acquisition Trust (AVAT) [48,50,64], the European Union (EU) COVID-19 vaccine joint procurement scheme (regional level) [11,32,34,47,66] ensured a centralized and coordinated procurement process securing COVID-19 vaccine access and equity for involved members. Other regional multilateral deals included the Gates Foundation, which worked with the Serum Institute of India (SII) and Gavi to accelerate the manufacturing and delivery of up to 100 million doses for LMICs in 2021 [30,74]. The Inclusive Vaccine Alliance (Germany, France, Italy, and the Netherlands) negotiated with AstraZeneca in August 2020 [69]. Local manufacturing in India [26,27], the USA [20,43], Australia [20,28,46], Denmark [52], Brazil [30,51], Germany [20], and the UK [20] made procurement of the vaccines relatively easy hence enabling availability at the health system level. South Korea [40,41] and Australia [46] swapped with other nations when vaccines were near expiry, ensuring continuous availability when vaccines would have been wasted. Donations of vaccines were made to COVAX and other countries by the HICs [28,44,52] and India [27].

LMICs had several procurement challenges, including the inability to manufacture vaccines due to lack of know-how, limited funding to purchase vaccines and technological capacity, no intellectual property rights (IPR), reliance on vaccine donations, and a lack of transparency on COVID-19 trials and regulatory hurdles, leading to reduced purchasing power with vaccine manufacturers and reduced vaccine access [59,64,67]. Tanzania and Ghana, for example, are the only African countries acknowledged by the WHO to have well-functioning regulatory systems for medicines [59]. Most LMICs relied on the COVAX facility, which had less power than some individual HICs [54]. Despite having better resources, manufacturing and regulatory capacities, and healthcare infrastructures than LMICs, some MICs (Brazil included) struggled with vaccine production, lack of transparency on COVID-19 trials, integration of health and industrial policy, low political will and politics surrounding the regulatory framework [30,51,60].

Scaling up production to meet the whole country’s demand and multiple doses needed to achieve immunity were challenging for HICs at the beginning of the pandemic [56]. The costs of distribution, administration, record keeping, and surveillance made vaccines unaffordable for many governments despite the income level from 2020 to early 2022 [56].

*Distribution and cold-chain capacity*

The factors that facilitated COVID-19 vaccine deployment included comprehensive cold-chain and distribution infrastructures for packaging, shipping, and storing mRNA vaccines (ultra-freezing fridges temperature monitoring tools), multiple fridges in various locations for vaccines and shelf-life monitoring applications, building a central vaccine storage site near the airport for easy distribution. The USA [43], Lebanon [55], New Zealand [55], Brazil [30], Israel [37], Bhutan [35], and South Korea [40,41] leveraged infrastructures built during the Influenza A virus pandemic in 2009. Vaccines were transported either by road in refrigerated vans or trucks, by domestic flights, and by helicopter services or drones to hard-to-reach places with no motor road access [26,33,55,59,65,66].

Deploying COVID-19 vaccines was challenging from 2020 to early 2022 due to the lack of access to ultra-freeze cold-chain infrastructure (mRNA vaccines) and the availability of multiple formulations that are outside the normal vaccines in national immunization program [20,27,28,30,33,38,58]. Additionally, managing the changes in shelf-life (due to the availability of new data) of the vaccines required the use of specialized tools to check expiration dates. This complicated vaccine inventory management [43,46]. Furthermore, the uncertain vaccine supply and significant variability in vaccine demand globally led to wastage [61]. In most HICs, this eased as more data became available [43].

At regional level, for example in Africa, challenges included a shortage of vehicles and fuel to move vaccines and vaccinators outside of urban areas, broken or outdated cold-chain equipment, short shelf-life, and efficacy concerns about Vaxzevria^®^ which led to vaccine disposal in Malawi [64], and the return of unused doses in South Sudan, hence wastage [64,67].

*Delivery models*

The use of delivery models differed from the usual fixed post-vaccination (family doctor clinics, hospitals or clinics, pharmacies); for example, mass vaccination, which was commonly adopted globally, facilitated reaching many people at once [28,31,44,45,49,50,51,55,57,66,67]. Churches, markets, sporting venues, parks, streets, nursing homes, and entertainment venues were used to support vaccination at scale.

Mobile vaccination was adopted in the USA [20,57], Germany [20,52,57], Australia [20,28], and Latvia [11] to reach a targeted population (for example, underserved minorities, hesitant, and marginalized populations) on a smaller scale.

Countries such as Israel [57], the UK [57], the USA [57], and some in the African region (e.g., Zambia, Sudan) [57,67] integrated the COVID-19 vaccine delivery with other health services routinely delivered, such as tuberculosis (TB), HIV, family planning antenatal clinics, schools with influenza vaccine programs, and blood donation camps in 2021.

The challenges with mass vaccination were costs for equipment, space, access, and operators [55]. Fixed post-vaccination requires medical and political leadership-led conversations to increase vaccine demand [57]. Integration of COVID-19 vaccines requires strong and coordinated existing services with infrastructure and workforce for it to be successful [57,67].

(4) **The outer setting domain of the CFIR**

*Policies and guidelines*

The WHO continuously updated its policies and guidance, which were adopted by many countries globally. Some of the guidance included the NDVPs [8], the WHO SAGE values framework for the allocation and prioritization of COVID-19 vaccination [75], and the WHO SAGE prioritization roadmap [12] (Supplementary Materials Table S2). The guidance helped in navigating supply challenges common in 2021 and protecting populations at risk of negative outcomes of COVID-19. 

Individual countries adopted and updated their vaccine prioritization policies according to the epidemiology, resources available, and vaccine supply. The groups prioritized varied per country but ranged from healthcare professionals, essential workers and frontline workers, the elderly, those with comorbidities and underlying health conditions, and staff and residents of residential care facilities [26,30,34,35,55,61,66,68]. Germany [52], Bulgaria [52], Denmark [52], South Korea [49], Japan [49], Italy [25], South Africa [25], India [25,26,36], and Singapore [49] and many countries [53] had free vaccination policies to increase vaccine uptake. There were also policies on the vaccine rollout elements of health workers [66], mass vaccination [55,68], procurement and distribution [31,53,68].

The main challenge was that new data emerged during the COVID-19 pandemic, which led to new and changed policies [31,44,67]. Implementation of these policies, which were sometimes in conflict with previous policies, led to vaccine hesitancy, confusion, lack of trust, fear of side effects, and delays to vaccine access and uptake [31,44,67,68]. For example, in Canada, the implementation of COVID-19 vaccine policies at different government levels was conflicting, leading to criticism of NACI [31]. Most LMICs were unable to make early plans or implement or update their guidelines due to low political will, compounded by lack of funding, leading to a delayed start of the COVID-19 vaccination program, high wastage, and untrained workers affecting deployment [10,53,59].

(5) **The inner setting domain of the CFIR**

*Organizations*

Organizations facilitated vaccine deployment and supply by providing structure, policies, leadership, and collaborations at all levels. At the global level, the COVAX facility funded some vaccine research and development, supporting and enhancing local expertise, procurement, allocation, and distribution of COVID-19 vaccines globally to ensure access, equity, and uptake [7].

At the regional level, different schemes emerged to collaborate on procurement, governance, and distribution. For example, the EU COVID-19 vaccine joint procurement scheme in the European region [11,25,32,34,47,66], AVAT in the African region [48,50,64], and PAHO RF in the Americas region [61].

The EU COVID-19 vaccine joint procurement scheme, a centralized EU procurement process comprised of 27 member states, aimed to secure sufficient production and access to vaccines and adapt the regulatory framework of its members according to the required urgency [76]. The AVAT, a centralized purchasing agent for the African Union (AU) Member States, aimed to secure the COVID-19 vaccines and financial resources for achieving Africa’s COVID-19 vaccination strategy targeted at 60% vaccine coverage of Africa’s population [77]. The PAHO RF, an old cooperation mechanism for the joint procurement of vaccines, syringes, and related supplies for participating members, was the designated procurement agency under the COVAX facility for all interested member states in the region of the Americas [78].

Organization-based barriers such as vaccine nationalism, an act of gaining preferential access to newly developed vaccines by individual countries [79], challenged vaccine availability and hence deployment as countries hoarded vaccines, leaving less for COVAX and regional organizations. The COVAX facility failed in its goal of delivering 2 billion vaccine doses by the end of 2021, with less than 50% of the original promise delivered [54,80]. This was attributed to vaccine nationalism, export restrictions by HICs amidst the pandemic, lack of vaccine production units in the Global South, limited technology transfer to other manufacturing partners to speed up production, dose sharing and dose donation problems, and dependency on AstraZeneca vaccine [54]. Other organizational challenges to vaccine deployment that COVAX facility and regional organizations faced were, for example, lack of participation and funding and lack of transparency regarding deals with pharmaceutical companies [54,58,59].

*Information, communication, and registration strategies*

The use of clear and open communication strategies, such as social listening and rumor tracking (a means of tracking conversations about a chosen topic or entity across social media platforms [81]), social media engagement, use of multiple languages, using multiple channels (toll-free telephone call centers, television, radio and bulletins from the official government), and provision of relevant vaccine information to the public, facilitated vaccine demand and vaccine uptake. This was seen in Europe [20,34,69], Africa [67], and India [61]. Communication of information was also conveyed at the implementer level among stakeholders, including health workers, to facilitate understanding of the substance, purpose, direction, and targets of the policy and goals. Furthermore, it ensured the updating of information as there were many changes in the vaccine rollout policies and processes [43].

Countries such as the USA [20,39], India [36], South Korea [41], Portugal [51], Andorra [11], Cyprus [11], Latvia [11], Luxembourg [11], Malta [11], Monaco [11], Montenegro [11], Slovenia [11], Israel [32], and Italy [32] used digital and non-digital registration systems for vaccine appointments enabling vaccine data surveillance. This data was used to continuously update resources and policies to facilitate vaccine deployment and uptake.

Vaccine uptake was hindered by confusing, ambiguous vaccine communication that was insensitive to language barriers and the education level of the public seen in some parts of Australia [46] and the USA [33]. Additionally, changing communication on the side effects of the AstraZeneca vaccine brought hesitancy and challenged uptake. In Africa, there were weaknesses in the capacity to respond to misinformation and media reports of alleged Adverse Events Following Immunization (AEFI) cases ahead of any official investigation and communication from officials [67]. In India [26] and the USA [33], vaccine uptake was hampered using online registration, which was challenging for those with limited digital literacy, e.g., some older adults or marginalized, hard-to-reach populations, some of whom were the prioritized targets for the vaccine.

*Existing healthcare systems*

According to the WHO, “a well-functioning health system is built on having trained and motivated health workers, a well-maintained infrastructure, and a reliable supply of medicines and technologies, backed by adequate funding, strong health plans and evidence-based policies” [82]. Germany, France, Italy, Finland, Switzerland, South Korea, Israel, Singapore, and Australia have strong public healthcare systems [83]. The COVID-19 vaccination programs in Canada [31], Israel [32,37], the USA [43,45], France [32], Italy [32], Spain [32], Germany [47], Austria [47], Switzerland [47], South Korea [40,41], Chile [29], and Australia [46] were supported by strong health systems characterized by good vaccine infrastructure built from the previous 2009 H1N1 influenza pandemic. Furthermore, countries updated COVID-19 policies and vaccination plans, had reliable vaccine supply and storage, well-coordinated leadership and partnerships among vaccine stakeholders, adequate funding for vaccine procurement, health workforce, and their motivation, good health plans and top-tier information systems, which facilitated high COVID-19 vaccine deployment and uptake [29,37,40,41,43,45,46]. 

Many LMICs lacked resources that strengthen healthcare systems, for example, health workers and training, a well-maintained infrastructure for the cold chain, a dependable and sustained supply of COVID-19 vaccines, adequate funding, strong health plans, and evidence-based policies hindering the deployment of vaccines [26,46,58,59,60,64,67]. HICs such as Australia [46] and Monaco [55], among others, struggled with poorly trained staff, poor governance, and logistics despite having a better pre-existing healthcare system. Brazil, a MIC, struggled with promoting vaccine delivery, partially due to medical and nursing workforce shortages and high demand for healthcare services despite a solid health infrastructure [30,51].

## 4. Discussion

The 50 articles included in this scoping review provide unique and timely insights into the healthcare system and policy factors that influenced COVID-19 vaccine deployment and uptake globally between December 2020 and 2023. The period from December 2020 to early 2022 was characterized by limited COVID-19 vaccine supply coupled with high vaccine demand. Healthcare systems leveraged and reformed existing infrastructures, some of which were built during the 2009 H1N1 pandemic, to maximize uptake. We have synthesized the evidence available and conclude that the hallmarks of successful vaccine deployment and uptake at the healthcare level are exemplified by the following eight characteristics: (i) the availability of updated and ready-to-use guidelines and policies for pandemic preparedness (deployment plans, the allocation and prioritization framework), (ii) a variety of procurement options, (iii) comprehensive cold-chain capacity and distribution strategies for each vaccine type (including mRNA vaccines), (iv) adequate resources including human resource and their continuous training (health workers and non-health workers to administer the vaccines), (v) the ability to implement a multitude of delivery models, (vi) strong leadership that leads a system that coordinates, plans, partners and communicates transparently and regularly to stakeholders, (vii) effective communication strategies, and lastly (viii) the state of the healthcare system itself.

Whilst many factors affect vaccine deployment and uptake, the wealth of a country undoubtedly has a major influence. HICs, for example, Israel, the USA, the UK, Canada, and Germany, had more successful uptake and deployment strategies than MICs or LMICs. This is potentially due to having more resources related to the affordability, availability, and accessibility of vaccine resources and infrastructure useful to build healthcare systems. Chen et al. report that high socioeconomic and health system-related factors played a key role in the COVID-19 vaccine rollout due to the availability of vaccination resources, hence predicting vaccine coverage [84]. Many HICs poured vast resources into COVID-19 vaccination development and availability to ensure speedy deployment and, thus, uptake in their vaccination campaigns [85,86].

Individual countries also used bespoke deployment strategies to increase COVID-19 vaccine uptake. These strategies, in many cases, were dependent upon available resources, and this was independent of country size, governments and policies, general healthcare state, economic state, leadership styles, healthcare organizational system, and manufacturing capability. Israel, for example, attributed its success to a unified strategy and centralized healthcare system, early pandemic preparation, a strong existing healthcare system, and its small population size, which enabled vaccine procurement, as collective bargaining, for the entire population [37,87]. On the other hand, the USA had no unified strategy but rather a decentralized system at the federal level and had the added challenge of a large population (ca. 340 million) [88]. Nonetheless, the USA was able to harness its strong existing healthcare system, early preparation, vaccine manufacturing capability, and experiences from previous influenza pandemics to succeed. Singapore and Bhutan’s high COVID-19 vaccine uptake has been attributed to the small population size and well-established healthcare systems [89,90,91]. This highlights the importance of a strong existing healthcare system, as well as coordination, planning, and funding for the vaccine deployment process.

The COVID-19 vaccination rollout process also highlighted the crucial role of countries’ governments and policymakers in vaccine deployment and uptake. Globally, bilateral deals (e.g., Pfizer and Swiss confederation, Israel, among others) [92,93] took precedence over multilateral deals (e.g., Advance Purchase Agreements (APAs) between AstraZeneca and the COVAX facility) [94] and those with better APAs were disproportionately at an advantage [95]. This is further evident in Latin American countries (e.g., Brazil and Argentina), which were at a disadvantage in procuring vaccines due to competing demands from Pfizer in February 2021 [96]. The impact of leadership, or lack thereof, was also evident in Brazil, whereby a lack of national leadership resulted in mayors and state governors having to take matters into their own hands. Despite Brazil’s lack of leadership, however, it still achieved high COVID-19 vaccine uptake, likely due to its consolidated immunization programs, decentralized primary care services, and expertise in global vaccine research, development, and manufacturing [51,97,98]. Some countries provided added value to manufacturers; for example, Israel offered to share the vaccine safety and efficacy data with Pfizer, which may have led to increased vaccine availability [93].

The healthcare workforce was a priority group as vaccine recipients and trusted professionals delivering vaccination, highlighting their integral role in vaccine deployment in various settings. Weintraub et al. report that healthcare workers have a role in COVID-19 vaccination education, counselling, and building trust [99]. Therefore, health workers should be engaged in planning, decision-making, policymaking, and communication strategies to increase their contribution to vaccine deployment.

Effective communication between stakeholders, the introduction of diverse communication tools that are data dependent on rumour tracking applications, and social listening to address concerning issues are required at the healthcare system level. A study on public responses to pandemic vaccination reported that providing safety information addressing vaccine misunderstandings and delivering mixed media campaigns across hospitals or communities improved vaccine uptake [100]. Countries should provide clear and continuous communication strategies and policies during pandemics and outbreaks to provide vaccine information and prevent vaccine misunderstandings.

At the policy and organizational levels, the COVAX facility initiative is progressive as a basic model for equitable, accessible, and affordable vaccines in pandemic times to achieve global health security and cooperation. However, as Pushkaran et al. argue, the COVAX current structure is inadequate to address the challenges the alliance faced and future pandemic challenges [54]. For instance, the pandemic exposed gaps in LMICs and MICs, such as the need for funding to support pandemic preparedness, including vaccine development and strengthening health systems. Additionally, sustainable solutions for vaccine supply, advocacy for sharing of IPR, technology transfers, and the importance of strong and sustained political will with reduced reliance on donations. Well-functioning regulatory systems are also essential. Global policies that address funding, technology transfers, and regulatory processes are urgently needed.

Our review findings capture and organize practical and complex healthcare systems issues to consider when deploying vaccines in an immunization campaign, in both pandemic and non-pandemic settings. The factors found in our review can be refined and included in a healthcare system framework for vaccine deployment and uptake globally, which is currently not present. Furthermore, future research is recommended to investigate the impact of the healthcare system factors of vaccination deployment and uptake on individual and population-level vaccine uptake rates. We note that there is no consistency in the vaccination nomenclature (at the healthcare system level) with different examples of the vaccine terms deployment, uptake, delivery, and rollout used across the studies and key documents. Hence, this can be studied further to work toward establishing a standardized nomenclature and improved reporting.

The COVID-19 pandemic exposed gaps in pandemic preparedness, particularly in vaccine deployment. Some studies were conducted on factors affecting COVID-19 vaccine rollout in a small setting (few countries or specific level of care) and specific elements of the vaccine rollout components including health system COVID-19 vaccine equity [19,20,55]. However, this review provides lessons learned from the different deployment strategies that were used globally to underpin future improvements to pandemic immunization campaigns. Additionally, as the COVID-19 vaccination was used for both adults and children, some of these strategies can be adopted in population-level vaccination programs to maximize vaccine deployment and uptake, as the WHO encourages life course immunization and as we aim to catch up with the Immunization Agenda 2030 [101]. For example, extending vaccine delivery to both secondary and tertiary level settings should be considered to increase points of contact to optimize vaccine delivery, as most routine vaccines are delivered at the primary care level. Continuous training of vaccinators and all healthcare workers as part of vaccine implementation has become complex due to different vaccine specifications and complex cold-chain requirements. Future pandemic vaccination programs should include comprehensive models that address the global vaccine deployment aspects promptly to achieve effective pandemic control. These include global financing of vaccination to ensure healthcare systems strengthening and global health governance, which the World Health Assembly 2024 committed to draft a global Pandemic Agreement by 2025 [102]. Models similar to the COVAX model should be used as a starting point. Regional and well-balanced research and development capabilities (e.g., IPR rights, technology sharing, transparency in the vaccine deals with the pharmaceutical industry, and regional clinical trials) are needed to support effective procurement, hence improving deployment. In addition, adequate and clear regulatory processes at the regional level, data-driven global and regional guidelines and policies, early pandemic plans that are sensitive to the uncertainty of events and mutations, sharing of data, effective communication strategies, equitable and centralized global and regional distribution and procurement models, and human resource management are vital. These factors can then be tailored at the national level according to the resources available. 

Our scoping review has many strengths. It was conducted with a robust and transparent methodology, conducting a comprehensive search in several databases. We included studies from various settings to obtain a global picture of health systems factors. We acknowledge that vaccine uptake success depends on multiple factors beyond just health systems, such as physical and psychological capability, social opportunity, and automatic and reflective motivation at the individual level, which should also be evaluated for effectiveness [88]. We recognize that the list of the included vaccine determinants might not be exhaustive or cover the full complexity of how various health system factors interact, as this information may not be presented in the published literature. We did not incorporate strict timelines for the COVID-19 vaccine rollout processes, as different countries had different timelines for vaccine deployment, and publication of information on deployment. As noted previously, the lack of a formal taxonomy or recognized definitions of terms such as deployment and uptake posed a challenge in developing the search strategy and ensuring consistency of interpretation across the studies included. 

## 5. Conclusions

COVID-19 vaccination deployment programs reformed their existing healthcare vaccination systems through strong coordination, leadership, and partnerships at the global (the COVAX facility), regional, and national levels. Furthermore, data-driven policies and innovative strategies such as the cold-chain and distribution capacity, different vaccine delivery models, use of a variety of vaccine platforms, prioritization of essential groups, early deployment plans, use of both digital and non-digital registration, and employing both health and non-health professionals to deliver vaccines were adopted to overcome limited vaccine infrastructure. Effective and transparent communication, implemented by healthcare systems, was used to dissolve misinformation and increase vaccine demand while adapting to uncertainty and changing times. This review provides the basic healthcare systems factors fundamental to vaccine deployment in immunization campaigns, and calls for the regrouping and building of healthcare systems frameworks for vaccine deployment to support future pandemic preparedness.

## Figures and Tables

**Table 1:** Healthcare system-based determinants of COVID-19 vaccine deployment and uptake globally grouped by the Consolidated Framework for Implementation Research (CFIR) [24].

CFIR Domain	Determinant of COVID-19 Vaccine Deployment and Uptake	Examples
**Innovation**	Types of vaccine products	(1) Use of a variety of new COVID-19 vaccine products (2) Use of mixed vaccination, whereby a different vaccine platform was used in subsequent immunizations
**Individuals**	Healthcare workforce capacity	(1) Employed both health and non-health professionals for COVID-19 vaccine deployment
	Partnership, coordination, and leadership	(1) Partnership between different stakeholders including government, NGOs, private organizations, and other stakeholders (2) Coordination: (i) Decentralized (COVID-19 vaccination program implementation was delegated away from the central government) (ii) Centralized (implementation was conducted by the central government) (iii) Mixed (implementation was conducted by the central government in collaboration with provincial and district or city governments) (3) Leadership and political will of policymakers, implementers and decision-makers
**Implementation process**	Procurement strategies	(1) Bilateral deals with vaccine manufacturers (2) Multilateral deals, i.e., COVAX facility (3) Donations from HICs, India (4) Vaccine swap with other countries (5) Procurement from local manufacturers
	Distribution and cold-chain capacity	(1) Infrastructures capable of ultracold chain shipping and storage capacity for mRNA vaccines and cold chain for other vaccines (2) Vaccine shelf-life extended as new stability data became available (3) Vaccine transportation and handling (4) Temperature monitoring
	Delivery models	(1) Fixed post-vaccination, e.g., primary care level clinics, hospitals (2) Mobile vaccination using vans to reach remote areas, use of drones (3) Mass vaccination in churches, markets, public spaces (4) Integration with other health services, e.g., hypertension/STI/HIV/antenatal clinics
**Outer setting**	Policies and guidelines	(1) **Global** Prioritization of at-risk groups (2) **National** National vaccine deployment plans Mandate vaccination Free vaccination
**Inner setting**	Organizations	(1) Global (COVAX facility) (2) Regional (EU COVID-19 vaccine joint procurement scheme, AVAT) (3) Local (country, state, or federal dependent organizations)
	Information, communication, and registration strategies	(1) **Open communication strategies including**: Social listening and rumor tracking Social media engagement and use of multiple languages and multiple channels Provision of relevant information to the public implementation stakeholders through Toll-free telephone call centers Television, radio National healthcare sources (2) **Registration** Use of digital and non-digital registration to book COVID-19 vaccination appointments (3) **Information** Vaccine surveillance data, inventory management, pharmacovigilance
	Existing health systems	(1) Infrastructure, supply of medicines and technologies, funding, strong health plans, health workers, evidence-based policies

Abbreviations: NGOs = non-governmental organizations; HICs = high-income countries; GP = general practitioner; STI = sexually transmitted infection; EU = European Union; AVAT = the African Vaccine Acquisition Trust; COVAX = the COVID-19 Vaccines Global Access Initiative.

## Data Availability

No new data were created or analyzed in this study; therefore, data sharing does not apply to this article.

## Supplementary Materials

The following supporting information can be downloaded at: https://www.mdpi.com/article/10.3390/vaccines12101093/s1, Table S1: An overview of the studies included in the review answering each objective; Table S2: Gray literature on COVID-19 vaccine policies adopted globally; Table S3: Preferred Reporting Items for Systematic Reviews and Meta-Analyses extension for Scoping Reviews (PRISMA-ScR) Checklist; Table S4: The search strategy.
