# Supplementary material for: A Scoping Review of Factors Affecting COVID-19 Vaccination Uptake and Deployment in Global Healthcare Systems
Source: Vaccines (Basel). 2024 Sep 25;12(10):1093. doi: 10.3390/vaccines12101093 (PMC11511325; doi:10.3390/vaccines12101093)
Supplement: Supplementary file 1 [file vaccines-12-01093-s001.zip › vaccines-3191501-supplementary.pdf]

Supplementary Materials

Table S1: An overview of the studies included in the review answering each objective

Setting, Author, publication year and type of study	Aim of study, with identified determinants of vaccine deployment presented as challenges and successes.
**Global studies**	
Global (6 WHO regions, India and Bahrain)<br>WHO 2021 [61]<br>Report	Aim: To summarize the discussions, conclusions, and recommendations for the COVID-19 immunisation program in 2021:<br><br>Challenges included the use of a variety of vaccine products and complexity, inadequate vaccine supply and funding, repurposing human resources management and training, conflict<br>Successes included cold chain and distribution capacity, multisectoral and multi-institutional partnerships, use of digital platforms, creation of vaccine demand and effective communication strategies, early planning COVID-19 national deployment and vaccination plans, policies on implementation, prioritization, service delivery, and information guidance, strong political commitment
Global (all countries)<br>Hasan 2021 [55]<br>Systematic review	Aim: To evaluate determinants of COVID-19 mass vaccination from Jan 2021 to 1 Mar 2021:<br><br>Challenges included inadequate vaccine supply, wastage, funding for vaccine supply, training, cold-chain, and other infrastructure requirements<br><br>Successes included existing health system, use of digital and non-digital registration of vaccine appointments, policies on vaccine prioritization, human resources (both health and non-health professionals)
Global (Australia, Hong Kong, Germany, France, Israel, Singapore, Spain, USA and the UK)<br>Aggarwal, 2022 [20]<br>Scoping review	Aim: To identify the barriers and facilitators to the vaccine roll-out:<br>Challenges included inadequate vaccine supply and delivery issues, cold chain and distribution capacity, shortage of health human resources, and poor communications<br>Successes included multifaceted vaccine distribution strategy, coordinated information systems, effective communications strategy and cultural awareness, leadership, partnerships, and collaboration with vaccination stakeholders, online registration of vaccine appointments, guidance documents with information on vaccination processes vaccine storage and distribution, policies on prioritisation, and workforce legislation
Global<br>Manriquez 2021 [60]<br>Article	Aim: To identify the access issues to COVID-19 vaccines and policy challenges regarding early phases of COVAX from Apr 2020 to May 2021:<br>Challenges included inadequate vaccine supply, procurement issues, lack of funding, lack of multilateral approach to global health and vaccine distribution, geopolitical conflicts, vaccine nationalism, access challenges to vaccines in MICs as they do not qualify for funding agencies (like GAVI) but are not rich enough to negotiate APAs, lack of cooperation at the global level.<br>Successes included use of a variety of vaccine products, vaccine production, intellectual property and technology transfer issues, transparency regarding vaccine deals and the safety and effectiveness of vaccines.

Global (all countries)<br>Khairi 2022 [58]<br>Review	Aim: To summarize the global efforts with respect to COVID-19 vaccine development, production, allocation, and deployment to achieve equitable access Dec 2020 to 1 Jul 2022:<br>Challenges in efforts included vaccine nationalism and procurement challenges by HICs threatened COVAX, inequity in the global vaccine distribution, inadequate vaccine supply, inadequate cold-chain and distribution capacities<br>Successes included global governance and strong leadership, policies to strengthen the manufacturing infrastructure and procurement capabilities
Global (185 countries)<br>Cameron-Blake, 2023 [53]<br>Article	Aim: To illustrate how the new dataset on COVID-19 vaccine prioritization, eligibility, distribution, cost and mandates can offer insights to researchers and policymakers from Jan to Jun 2022:<br>Vaccine costs and mandates: Most countries provided the vaccine free of charge with few countries Botswana, India, Pakistan and Turkmenistan offering them on a small fee. Vaccine mandates were not universal in all countries.<br>Most countries adopted policies on prioritization, eligibility and deployment plans to maximise constrained vaccine supply
Global (all countries)<br>Wouters [56]<br>Health Policy	Aim: To review potential challenges to success of COVID-19 vaccines in each of these dimensions (development and production, affordability, allocation and deployment) and discuss policy implications in 2021:<br>Challenges included vaccine production manufacturing capacity, scaling up production to meet global demand, inadequate global supply chains for inputs, such as glass vials, syringes, and stabilising agents, inadequate vaccine supply, funding, vaccine nationalism, logistical and administrative challenges i.e. data, cold chains and transportation, Healthcare worker issues, short time available for national, regional, and local health officials to plan training and preparedness for COVID-19 vaccination programmes<br>Successes included policies on vaccine prioritisation, collaboration and partnerships between stakeholders
Global ((USA, Italy, Malta, Israel, Germany, England, South Africa, China, Sudan)<br>Nabia 2023 [57]<br>Rapid review	Aim: Look at experiences on COVID-19 vaccine service delivery and integration to inform future COVID-19 vaccination programming (into immunization programs and primary healthcare services) from Dec 2020:<br>Successes included the use of various vaccine delivery models, multi-sectoral partnerships among stakeholders, adequate cold chain and distribution capacity, maintenance of data systems and appointment systems, policies on vaccine prioritisation, adequate vaccine supply in HICs than LMICs, engaging both health and non-health professionals in vaccine delivery<br>Challenges in planning and coordination and securing adequate human resources, logistical constraints in vaccine storage and transportation due to in adequate infrastructure, an inequitable spread of health facilities, faulty digital appointment systems in optimizing appointment slots
Global (all countries)<br>Privor-Dumm 2023 [59]<br>Article	Aim: To look at equitable access and whether COVAX ensured that vaccines were distributed equitably till 2022:<br>Challenges to access included inadequate vaccine supply, lack of collaboration among countries globally to increase vaccine access and equity, waning vaccine demand, lack of sustained political will and funding to support vaccine development<br>Successes included policies on prioritization, procurement and distribution, and mandate requirements

Global (all countries)<br>Pushkaran, 2023 [54]<br>Review	Aim: To identify the implementation challenges and framework gaps of COVAX and to explore the interconnected global health policy and governance gaps in ensuring equity, accessibility and affordability of vaccines from 2020 to 2022:<br><br>Challenges included vaccine nationalism export restrictions by wealthy countries, lack of HICs participation and funding for COVAX, lack of transparency in the vaccine deals with pharma companies, lack of vaccine production units in the Global South and not enough technology is transferred to other manufacturing partners to speed up production, dose sharing and dose donation problems, lack of global health governance mechanisms to control the decision-making power
Italy, India, South Africa<br>Yang 2022 [25]<br>Article	Aim: To analyse and to provide empirical experience for COVID-19 vaccine policies and their effectiveness in Italy, India, and South Africa till April 2022:<br>Challenges included inadequate vaccine supply, vaccine production, procurement issues, weak existing healthcare system, inadequate Cold chain and distribution capacity, lack of funding.<br>Successes included online vaccine registration of vaccine appointments, policies on vaccine prioritisation, vaccine appointments, free vaccination, vaccination plan, mandatory vaccination.
**Low- or Middle-Income Countries studies**	
LMICs<br>Peacocke 2021 [10]<br>Review	Aim: To identify the factors contributing to equitable access to COVID-19 vaccines for LMICs from Aug 2020-May 2021:<br><br>Challenges included vaccine development, local production and technological capacity, reduced procurement power and funding, regulatory delays weak existing health system, inadequate cold chain and distribution capacity and geopolitics<br><br>Success included collective or pooled procurement
Africa<br>Masresha 2022 [67]<br>Review	Aim: Identify the key lessons and challenges of COVID vaccine roll out in 22 African countries in 2021:<br>Challenges included inadequate vaccine supply, weak existing health system hence poor balance of delivery COVID-19 vaccine and routine vaccines, poor Coordination and collaboration with vaccination stakeholders, lack of funding, poor cold chain and distribution capacity, challenges associated with the introduction of multiple types of new vaccines with short shelf life into national immunisation programs in a short period of time, shortage human resources Success included effective communications strategy
Bhutan<br>Phuntsho, 2022 [35]<br>Article	Aim: To share Bhutan's experiences on conducting the nationwide COVID-19 vaccination rollout from Jul 2020 to Jun 2022:<br><br>Successes included adequate cold chain and distribution capacity, early planning for COVID-19 vaccination that was updated according to resource availability (cold chain assessment, human resource mapping, electronic recording and reporting system for real-time vaccination coverage, and AEFI monitoring), policies on vaccine deployment plans and prioritisation, adequate human resources and training, adequate vaccine supply, the Bhutan Vaccine System (BVS) which ensured quality data collection for evidencebased planning, existing strong foundation of a national immunization program and experiences in conducting the nationwide vaccination campaigns in the past, digitalised appointment system, great leadership<br><br>Challenge included vaccine wastage

India<br>Gulati, 2022 [26]<br>Review	Aim: To describe challenges to scale up India's vaccination campaign and addresses strategies in 2021:<br><br>Challenges included centralized control of the vaccination programme, poor cold chain and distribution capacity, failure to reform public health, poor leadership and vaccine politics, scarcity of human resources, Online registration of vaccine appointments, limited vaccine procurement and manufacturing capability<br><br>Successes included policies on vaccine prioritisation, ability to import foreign vaccines to increase vaccine supply
Africa<br>Ayenigbara, 2021 [64]<br>Review	Aim: To describe challenges to COVID-19 vaccination programme in Africa from Jan to Jul 2021:<br><br>Challenges included limited funding and low political will, scarcity of the vaccine, inadequate cold chain and distribution capacity, poor human resources management and training, poor existing health system, use of a variety of vaccine products challenges such as poor tracking and records of specific vaccines, reliance on GAVI and external donors and bodies, wars and conflicts-hard to reach<br><br>Successes included organisation and coordination, pooled vaccine procurement by the AU
Senegal<br>Desclaux, 2024 [50]<br>Qualitative study	Aim: To explore how vaccination was implemented and experiences with the preparedness process, for making Senegal ready for COVID19 vaccination from 2020 to April 2022:<br><br>Successes included policies on vaccine prioritisation, use of experiences and expertise from the Ebola epidemic, Immunisation plans covering coordination, financing, legislation, logistics and cold chain, pharmacovigilance evaluation, and demand generation, use of a variety of vaccine products<br><br>Challenges included inadequate vaccine supply, uncertainty on the relevance of vaccination strategy, regulatory and reallocation issues, difficulties in complying with 2 doses, low vaccine demand at the end when supply issues were addressed, inadequate pharmaceutical research and production of biomedical equipment, vaccine transport and storage challenges, shortage of human resources
Ghana<br><br>Abraham, 2023 [48]<br><br>Article	Aim: To explore the perspectives of vaccinators on the health system factors impacting the COVID-19 vaccination campaign from 1 March 2021 to 31 March 2023:<br>Successes included policies on vaccine prioritisation, use of a variety of vaccine products, human resources and training before vaccine campaigns, use of existing immunization programs, evaluation, and demand generation, data surveillance, effective communication strategies, strong partnerships, coordination and leadership<br>Challenges included inadequate vaccine supply, inadequate cold chain and distribution capacity, lack of motivation and inadequate human resources, difficulties in locating convenient areas for vaccination campaigns
India<br>Purohit, 2022 [36]<br>Article	Aim: To present a comprehensive overview of COVID-19-related measures, largely centred around the development of vaccination related policies, their implementation and challenges faced in the vaccination drive in India from 2020 to 2021:<br>Successes included collaboration with vaccine stakeholders, use of digital systems to register vaccine appointments, early preparation of operational guidelines and policies on procurement, liberalized vaccination policy, vaccine prioritisation, and allocation and the monopoly of central government in vaccine procurement<br>Challenges included inadequate vaccine supply and implementation of liberalised policies on vaccine pricing

India<br>Mathur, 2022 [27]<br>Article	Aim: To analyse India's COVID-19 vaccination policy by the problem, politics and policy model during the pandemic in 2021:<br><br>Challenges included inadequate vaccine supply and lack of coherent policies on procurement and distribution, and vaccine politics<br><br>Successes included vaccine diplomacy, policies on vaccine prioritisation
**Middle Income Countries studies**	
Brazil<br>Fonseca, 2021 [30]<br>Article	Aim: To outline lessons (challenges and opportunities) from Brazil (MIC) COVID-19 vaccination; procurement and production; regulation of marketing registration; and distribution and uptake:<br><br>Challenges included inadequate vaccine supply, inadequate cold chain and distribution capacity, poor political commitment, political will and leadership, disputes, legal and technical problems hampered agreements for technology transfer and local production, competition dose procurement with HICs, lack of transparency on COVID-19 vaccines trials<br><br>Successes included policies on vaccine prioritization, diversification on where to get COVID-19 vaccines, purchases with agreements for technology transfer and local production, historical alignment of the health system with production facilities
Portugal and Brazil<br>Sequeira, 2023 [51]<br>Article	Aim: a retrospective analysis of the impact of COVID-19 vaccines on health and socioeconomic outcomes in Finland in the endemic phase of the COVID-19 pandemic from 2020 to Apr 2023<br>Successes included policies on vaccine prioritization, appointment and voluntary vaccination, strong collaboration between institutions and organizations, consolidated immunization programs and decentralized primary care services and health systems, domestic production of vaccines, efficient and available vaccination centres, higher trust in government in Portugal<br>Challenges included Low political will in Brazil to implement the COVID-19 vaccination program and procure vaccines, shortage of medical stuff in Portugal and strained healthcare systems in both countries
**High-Income Countries studies**	
Europe<br>Ares-Blanco, 2021 [66]<br>Article	Aim: To describe the response of different EU countries to the strategic challenges of guaranteeing human resources in PC and the implementation of the vaccination campaign against SARS-CoV-2 in 2021:<br><br>Successes included policies on prioritization, working conditions, incentives, procurement and transportation, human resources management and training, coordination and collaboration among stakeholders
Chile<br>Castillo, 2021 [29]<br>Article	Aim: To identify the elements behind Chile's successful COVID-19 vaccine rollout from 2020 to 2021:<br><br>Successes included vaccine availability, the centralized government and partnerships with stakeholders, leveraging existing healthcare system and experience from H1N1 pandemic, provision of human, technical and local resources, existence of an electronic vaccine registry, designed strategy which considered capacity of the existing systems

France, Israel, Italy and Spain<br>Antonini, 2022 [32]<br>Article	Aim: To look at the COVID-19 vaccination campaigns in France, Israel, Italy and Spain during the first eleven months from the first COVID19 vaccine approval from Dec 2020 to Nov 2021: Challenge included inadequate vaccine supply
	Successes included coordination and collaboration among stakeholders, good leadership, adequate human resources and training, early procurement agreements, effective Information systems, policies on vaccine prioritization, compulsory vaccination for selected groups, people's trust in the public health system, effective cold chain and distribution capacity
Canada<br>AlShurman, 2023 [31]<br>Policy analysis	Aim-To examine the first year of Canada's COVID-19 vaccine program to identify challenges and lessons learned for vaccination policies from 1 Oct 2020 to 1 Dec 2021:<br><br>Challenges included inadequate vaccine supply and complicated vaccine schedules, frequent vaccination policy changes were confusing people shaking their confidence, decentralization of multi-level governance was confusing people, vaccine mandates led to ethical protests of autonomy<br><br>Successes included collaborations and partnerships among stakeholders, policies on dose interval, vaccine schedules and mandates, and procurement, strong existing health systems introduction he mix-and-match policy (those who had a first dose of AstraZeneca to complete their vaccine series with an mRNA vaccine), frequent update of guidelines according to evidence available
Canada<br>Kholina 2022 [38]<br>Article	Aim: To examine the COVID-19 vaccine rollout in Canada through the lens of vulnerability, focusing on vaccination strategies and tactics adopted in Alberta, Ontario, Nova Scotia, and Yukon:<br>Challenges included inadequate vaccine supply, inadequate cold chain and distribution capacity, technological, and language barriers<br>Successes included adequate human resources management and training, vaccine appointments system and policies on vaccine prioritization
South Korea<br>Hong 2022 [40]<br>Article	Aim: To help identify the critical attributes that allowed the successful rollout of the COVID-19 vaccine and new insights of relevance to public health practitioners and planetary health policy scholars from Jan 2021 to 31 Mar 2022:<br>Challenge included inadequate vaccine supply in the initial stages<br>Successes included centralized hierarchical administrative system with a strong state capacity, leadership collaboration among different ministries to implement speedy crisis management, guidelines for local governments, municipalities, and other health units, vaccine advocacy by top government officials; Prime Minister, The President, Korean ministers, adequate cold chain and distribution capacity, human resources management and training, prior experience from MERS-CoV outbreak response, local production and vaccine swapping, strong existing health system, existing successful and accessible public health systems, policies on delivery, accessibility, and prioritisation
South Korea<br>Kwon and Oh 2022 [41]<br>Article	Aim: An overview of South Korea's COVID-19 vaccination program and the key measures the country enacted to overcome the initial vaccine shortage and expand the vaccinated population from Dec 2020 to Aug 2021:<br>Challenges included inadequate vaccine supply, reluctance to implement the COVID-19 vaccination in early pandemic times, and socioeconomic disruption<br>Successes included strong partnerships among vaccine stakeholders, policies on vaccine prioritisation, strong pre-existing universal health care system, COVID-19 vaccination plan and data driven revision of the plan with transparency, use of technologies, previous experience with the MERS epidemic, adequate vaccine supply in later stages

Europe<br>Rob Van Kessel 2023 [68]<br>Article	Aim: To perform an in-depth analysis of the active COVID-19 vaccine policies in effect across Europe and capture policy divergences between European countries from Jan 2022 and Apr 2022:<br>Policies on vaccine authorization, prioritization, procurement and distribution, data collection, administration, and mandate requirements<br>which were slightly different among the European countries, incremental access to the vaccines despite initial supply shortages

Finland<br>Tiirinki 2022 [34]<br>Article	Aim: To analyse the vaccination strategy as part of wider public governing of the COVID-19 pandemic in Finland from Mar 2020 to Dec 2021:<br><br>Challenges included inadequate vaccine supply, complicated vaccine logistics due to cold storage, self-request registration of vaccine appointments<br><br>Successes included strong partnerships among vaccine stakeholders, policies and recommendations on procurement, dosing interval, and vaccine prioritisation, clear and open vaccine communication
Europe<br>Palmer 2022 [11]<br>Article	Aim: To describe the challenges that WHO Small Countries Initiative (SCI) countries faced in procuring vaccines, setting up vaccination sites, and administrating vaccines to different priority groups; describe the systems used for vaccine registration and vaccination efforts and share the methods that the countries used to address problems in 2021:<br><br>Challenges included inadequate vaccine supply at the beginning of roll out, procurement challenges, vaccine wastage, use of digital booking systems which were difficult to access by some high-risk populations, shortage of human resources, lack of appropriate vaccination centres<br><br>Successes included use of different vaccine delivery model, effective communication strategies, presence of a national deployment and vaccination plan and policies on vaccine prioritisation and procurement, clear and open vaccine communication and use of both health and non-health professionals for vaccination
Israel<br>Rosen B 2021 [37]<br>Article	Aim: An overview of the Israeli rollout, and the 12 factors that contributed to its early success:<br><br>Successes included adequate cold chain and distribution capacity, early planning of the roll out program, early regulatory approval following FDA, small population size, collaboration and organisation among stakeholders, human resources management and training, Israel's centralized national system of government, Israel's experience in, and for, planning and implementing prompt responses to large-scale national emergencies, policies on vaccine prioritisation and procurement, strong existing health system, well-tailored vaccine demand efforts, existence of frameworks and support tools for implementing vaccine decisions, adequate funding and early contracting for large amounts of vaccines
Eastern Mediterranean region<br>Hasan 2022 [63]<br>Article	Aim: To identify bottlenecks in access, deployment efforts, and use of COVID-19 vaccines in the EMR and propose recommendations for improving vaccine coverage from Jun to Jul and Oct–Nov 2021:<br><br>Challenges included inadequate vaccine supply, regulatory approval issues (accelerated pace), vaccine delivery issues, weak existing health in some countries, inadequate cold chain and distribution capacity

Australia<br>Jayasinghe, 2023 [28]<br>Review	Aim: To describe the role of the ATAGI in the national COVID-19 vaccination program, in terms of the initial programmatic recommendations in the evolving context of evidence relating to the disease and vaccines, and the program rollout in 2021:<br><br>Challenges included inadequate vaccine supply in the first six months, vaccine use in outbreak settings, inadequate workforce capacity, poor distribution and access logistics
	Successes included policies on vaccine prioritisation, and partnership with vaccine stakeholders
Europe<br>Vogler, 2021 [65]<br>Article	Aim: To describe the experiences of the countries' collaborations such as the Nordic Pharmaceutical Forum (involving Denmark, Iceland, Norway and Sweden, with Finland as an observer) and the Baltic Procurement Initiative- (Estonia, Latvia and Lithuania,) the Beneluxa Initiative on Pharmaceutical Policy (involving Austria, Belgium, Ireland, Luxembourg and the Netherlands) in 2021:<br>Vaccine procurement issues including pooled procurement to improve access due to higher purchasing power, use of existing knowledge and the experience gained during previous procurement processes
USA<br>Moss 2022 [33]<br>Article	Aim: To identify successes and failures of COVID vaccine distribution and provide a best practice framework for future emergency responses including mass vaccinations:<br><br>Successes included provision of vaccine information to people, online vaccine registration of vaccine appointments, policies on vaccine prioritisation and distribution<br><br>Challenges included inadequate vaccine supply, confusing communication between the three levels of government, federal, state and county governments, poor leadership and political will by the president, inadequate cold chain and distribution capacity, vaccine delivery issues, confusing vaccine communication from the federal leaders
USA<br>Pammal 2021<br>[42]<br>Policy Analysis	Aim: To highlight the importance of including the Federal Retail Pharmacy Program, in the national COVID-19 vaccine distribution plan to ensure equitable vaccine distribution and access:<br>Successes included policies on vaccine prioritisation, and distribution plan, use of federal government capabilities and resources to ensure equity in vaccine distribution, engaged in the federal pharmacy partnership strategy, distribution models that effectively involve pharmacists and information and awareness campaigns to ensure public trust<br>Challenges in developing comprehensive plans that determine priority populations and immunization registries
USA<br>Shen 2022 [45]<br>Review	Aim: To examine immunization information systems and the role that data and staffing play in the context of the COVID-19 pandemic response and the policies and practices to respond to the COVID-19 public health emergency in 2021:<br><br>Successes included COVID-19 vaccination strategy was built on existing vaccination infrastructure, leveraged and strengthened partnerships that were created during the H1N1 pandemic among stakeholders, bidirectional data exchanges, data reporting and analysis with other jurisdictions

Australia<br>Gillepsie 2022 [46]<br>Article	Aim: To look at Australia's paradoxical situation of low and high COVID-19 vaccination rate between mid to late 2021:<br><br>Successes included policies on vaccine prioritisation and national deployment plan, use of existing systems and governance mechanisms as the basis of the response, experience from prior effective seasonal influenza immunization programs, use of established existing healthcare systems, use of both health and non-health professionals in vaccine implementation, Different vaccine delivery models, adequate cold chain and distribution capacity in the later stages<br><br>Challenges included inadequate vaccine supply, reliance on domestic production and a preference for untried private sector distribution channels inadequate human resources and training, Procurement issues, poor communication (confusing and ambiguous)
Germany, Austria and Switzerland<br>Desson, 2022 [47]<br>Article	Aim: To understand the factors that influenced the DACH countries' (Germany (D), Austria (A) and Switzerland (CH) deployment of COVID-19 vaccines and key lessons to be learned from the region:<br>Successes included policies on mandatory vaccination and prioritisation, healthcare systems all three countries were also successfully able to adapt to the increased demands presented by COVID-19, effective capabilities regarding vaccine administration, leadership and governance systems<br>Challenges included lack of transparency throughout the procurement processes inadequate vaccine supply during the early stages of their immunization campaigns
Europe<br>Cadeddu, 2022 [69]<br>Article	Aim: an overview of aspects related to the planning and organization of COVID-19 vaccination campaigns in its initial eight months in a group of EU countries from different subregions of Europe from Dec 2020 to Aug 2021:<br><br>Successes included clear vaccination strategy, national immunization plans and policies on free vaccination and prioritisation, coordination among vaccine stakeholders, communication strategies, creation a procurement Alliance "Inclusive Vaccine Alliance"
USA<br>Bloomstone, 2022 [39]<br>Review	Aim: To synthesize LHDs' COVID-19 vaccine allocation and administration processes, identify successes and challenges faced, and highlight lessons learned to improve future mass vaccination campaigns from Dec 2020 to 3 July 2021:<br><br>Successes included online vaccine registration system, policies on vaccine prioritization, use of both health and non-health professionals in vaccine implementation, use of expertise and lessons learnt from previous vaccination.<br><br>Challenges included inadequate vaccine supply, inadequate funding for human resources and training, low political will and politicisation of the process and strategies of the COVID-19 response, challenges in creating and maintaining partnerships complex cold chain and distribution requirements, lack of local input in decision making as LHDs' were not included

USA<br>Duggar, 2024 [43]<br>Article	Aim: To describe the challenges posed by COVID-19 vaccines concerning prior pandemic planning and the 2009 H1N1 influenza pandemic experience, and the infrastructure supporting the distribution of COVID-19 vaccines. lessons learned regarding the future planning of emergency response vaccine distribution efforts from Dec 2020 to May 2023:<br>Successes included Leveraging existing infrastructure for VFC, collaboration, and partnership, with state and local health departments, distributors, manufacturers, multiple federal agencies, and vaccine providers, communication between the partnerships, utilisation and improvements in the utilization IIS for routine immunizations to use for COVID-19 vaccines information exchange on distribution supply and wastage, policies on mandatory vaccination, vaccine procurement and supply, vaccine appointment and prioritisation
Korea, Japan, and Singapore<br>Ma, 2023 [49]<br>Article	Aim: To analyse COVID-19 vaccine policies and effectiveness in Korea, Japan, and Singapore:<br>Successes included clear communication strategies and vaccine information widely available to the public, provision of a vaccine booking system, collaboration between the government, medical institutions and community, efficient operations and strict enforcement of regulations, vaccine supply, policies on vaccine prioritisation, appointments, costs, incentives, mandatory vaccination, procurement and supply, different vaccine delivery models, high political will, adequate vaccine supply when vaccine scarcity was no longer an issue<br>Challenges included inadequate vaccine supply initially, lack of Funding for vaccine research and development
Finland<br>Tiirinki, 2024 [44]<br>Article	Aim: a retrospective analysis of the impact of COVID-19 vaccines on health and socioeconomic outcomes in Finland in the endemic phase of the COVID-19 pandemic:<br>Successes included policies on vaccine prioritisation, sufficient state capacity, effective communication strategies, strong formal political institutions, health, and social policies to support the compliance of the people<br>Challenges included inadequate vaccine supply, changing vaccine strategies due to change in vaccine data, lack of integrity in terms of its COVID-19 research and policy recommendations
Germany, Denmark and Bulgaria<br><br>Xie, 2024 [52]<br><br>Article	Aim: To examine the COVID-19 vaccine policies disparities and effectiveness in Germany, Denmark and Bulgaria, with a view to providing lessons for global vaccination and response to outbreak risks:<br>Challenges included ill-prepared health (in crisis), human resources shortage, mistrust in government<br><br>Successes included Policies on vaccine prioritisation, appointment and free vaccination, procurement and supply<br><br>Despite the EU's uniform to ensure equitable distribution of vaccines among EU countries, there are differences in vaccination conditions, effectiveness, and types of responses to vaccine safety issues among the three countries

Abbreviations: Gavi Global Alliance for Vaccines and Immunisation, PC primary care, H1N1 Influenza Influenza A virus subtype H1N1, MERS-CoV Middle East Respiratory Syndrome coronavirus, AEFI Adverse Events Following Immunisation, APAs Advance Purchase Agreements, LMICs Lower Middle-Income Countries, MICs Middle-Income Countries, HICs High-Income Countries, AU African Union, IIS Immunization Information Systems, VFC Vaccines for Children Program, LHDs local health departments, EU European Union, Australian Technical Advisory Group on Immunisation (ATAGI), FDA Food and Drug Administration

Table S2: Grey literature on COVID-19 vaccine policies adopted globally

Title	Author, date	Type of document	Setting	Aim	Recommendations
Guidance on developing a national deployment and vaccination plan for COVID-19 vaccines: interim guidance [8]	WHO, June 2021	interim guidance	Global	to guide national governments in developing and updating their national deployment and vaccination plan (NDVP) for COVID-19 vaccines	Things to consider when making a deployment plan include<br>1. Types of COVID-19 vaccines and variety of approaches<br>2. Health system strengthening, including strengthening immunization systems through (A well-performing or improving and responsive immunization programme)<br>3. Coordination with other health programmes or sectors<br>4. Regulatory preparedness<br>5. Costing and funding: ensuring funds reach the point of deliver<br>6. Identifying and prioritising target populations<br>7. Vaccination delivery strategies<br>8. Preparation of supply chain and management of health care waste<br>9. Human resource management and training<br>10. Vaccine acceptance and uptake (demand)<br>11. immunization monitoring systems<br>12. 13. Evaluation of COVID-19 vaccine introduction

Guidance on operational microplanning for COVID-19 vaccination [62]	WHO, Nov 2021	interim guidance	Global	To provide operational guidance and information to support planners and immunization programme managers at the national and sub-national levels on COVID-19 vaccination implementations	Challenges associated with COVID-19 vaccination – which vary according to context and vaccine availability:<br>1. age of high-risk groups and vulnerable individuals outside the routine immunization system<br>2. erratic and unpredictable supply<br>3. short shelf life, complex vaccine handling and stringent storage requirements for some vaccines<br>4. management of multiple vaccine products with differing vaccine efficacy, target age groups and recommended schedules and novel vaccine presentations<br>5. limited and rapidly evolving information on the vaccine supply, future immunization schedule<br>6. limited human resources<br>7. urgency to deliver vaccine during a crisis, when health systems are strained.<br>8. communications about vaccines and managing misinformation, rumors and other concerns that can impede uptake and confidence<br>9. management of expectations and preferences when vaccine supply is constrained while motivating people to get vaccinated in places where vaccines are available<br>components necessary for management of human resources, vaccines and logistics.
					demand generation and communications; service delivery; and community engagement include.<br>• Determine target, estimate requirements plan vaccine storage, identify and manage human resources, plan service delivery, generate demand & ensure communications, monitor implementation, re-evaluate plan

WHO SAGE roadmap for prioritizing<br>Uses of covid-19 vaccines in the Context of limited supply [12]	WHO, Nov 2020	interim guidance	Global	To assist in developing recommendations for use of vaccines against COVID-19 based upon<br>epidemiologic setting and vaccine supply scenarios.	Guidance of on three scenarios of constrained vaccine supply were given under various contingencies<br>Stage I very limited vaccine availability (ranging from 1–10% of a country's total population) for initial distribution<br>Stage II scenario as vaccine supply increases but availability remains limited, (ranging from 11–20% of a country's total<br>population)<br>(Stages I and II correspond to the Phase 1 supply of the COVAX Allocation- up to 20% of each country's population)<br><br>Stage III scenario as vaccine supply reaches moderate availability (ranging from 21–50% of a country's total population).<br>Stage III scenario aligns with the COVAX Allocation Framework's Phase 2 supply of more than 20% population coverage.<br>Contingencies (plausible assumptions regarding vaccine characteristics, clinical and epidemiologic importance) under which the roadmap prioritisation criteria should be changed or unchanged included:<br>1. Number and timing of vaccine doses and vaccine types<br>2. Vaccine uptake, efficacy and safety<br>3. Epidemic conditions and immune status<br>4. Social, Economic and Legal Contexts

Table S3: Preferred Reporting Items for Systematic reviews and Meta-Analyses extension for Scoping Reviews (PRISMA-ScR) Checklist

SECTION	ITEM	PRISMA-ScR CHECKLIST ITEM	REPORTED ON PAGE #
**TITLE**			
Title	1	Identify the report as a scoping review.	1
**ABSTRACT**			
Structured summary	2	Provide a structured summary that includes (as applicable): background, objectives, eligibility criteria, sources of evidence, charting methods, results, and conclusions that relate to the review questions and objectives.	1

SECTION	ITEM	PRISMA-ScR CHECKLIST ITEM	REPORTED ON PAGE #

**INTRODUCTION**			
Rationale	3	Describe the rationale for the review in the context of what is already known. Explain why the review questions/objectives lend themselves to a scoping review approach.	2-3
Objectives	4	Provide an explicit statement of the questions and objectives being addressed with reference to their key elements (e.g., population or participants, concepts, and context) or other relevant key elements used to conceptualize the review questions and/or objectives.	3
**METHODS**			
Protocol and registration	5	Indicate whether a review protocol exists; state if and where it can be accessed (e.g., a Web address); and if available, provide registration information, including the registration number.	3
Eligibility criteria	6	Specify characteristics of the sources of evidence used as eligibility criteria (e.g., years considered, language, and publication status), and provide a rationale.	3
Information sources*	7	Describe all information sources in the search (e.g., databases with dates of coverage and contact with authors to identify additional sources), as well as the date the most recent search was executed.	3-4
Search	8	Present the full electronic search strategy for at least 1 database, including any limits used, such that it could be repeated.	Supplementary material 4
Selection of sources of evidence†	9	State the process for selecting sources of evidence (i.e., screening and eligibility) included in the scoping review.	4
Data charting process‡	10	Describe the methods of charting data from the included sources of evidence (e.g., calibrated forms or forms that have been tested by the team before their use, and whether data charting was done independently or in duplicate) and any processes for obtaining and confirming data from investigators.	4
Data items	11	List and define all variables for which data were sought and any assumptions and simplifications made.	4
Critical appraisal of individual sources of evidence§	12	If done, provide a rationale for conducting a critical appraisal of included sources of evidence; describe the methods used and how this information was used in any data synthesis (if appropriate).	NA
**Synthesis of results**	**13**	Describe the methods of handling and summarizing the data that were charted.	**4**
**RESULTS**			
Selection of sources of evidence	14	Give numbers of sources of evidence screened, assessed for eligibility, and included in the review, with reasons for exclusions at each stage, ideally using a flow diagram.	5
Characteristics of sources of evidence	15	For each source of evidence, present characteristics for which data were charted and provide the citations.	5
Critical appraisal within sources of evidence	16	If done, present data on critical appraisal of included sources of evidence (see item 12).	NA

Results of individual sources of evidence	17	For each included source of evidence, present the relevant data that were charted that relate to the review questions and objectives.	Supplementary material 1
**Synthesis of results**	**18**	Summarize and/or present the charting results as they relate to the review questions and objectives.	**6**-13
**DISCUSSION**			
**SECTION**	**ITEM**	**PRISMA-ScR CHECKLIST ITEM**	**REPORTED ON PAGE #**
Summary of evidence	19	Summarize the main results (including an overview of concepts, themes, and types of evidence available), link to the review questions and objectives, and consider the relevance to key groups.	13-15
Limitations	20	Discuss the limitations of the scoping review process.	16
Conclusions	21	Provide a general interpretation of the results with respect to the review questions and objectives, as well as potential implications and/or next steps.	14-15
**FUNDING**			
Funding	22	Describe sources of funding for the included sources of evidence, as well as sources of funding for the scoping review. Describe the role of the funders of the scoping review.	16

Table S4: The search strategy

**Research Topic**	A scoping review of the healthcare system-based factors affecting COVID-19 vaccination deployment and uptake within healthcare systems globally.
**Search Strategy Key concept**	**Synonyms/alternative terminology – combine using OR**
**SARS-CoV-2 vaccine**	Coronavirus Disease 2019 vaccine OR<br>covid-19 virus immunization OR sars-<br>cov-2 immunisation OR sars-cov-2<br>immunization OR<br>sars-cov-2 vaccine OR covid-19 immunization or covid-19 immunisation OR covid-19 virus vaccination OR covid-19 virus immunisation OR<br>Coronavirus Disease 2019 vaccination

**healthcare system-based factors**	Vaccine campaign OR Vaccine policy OR<br>Vaccine rollout OR Vaccine implementation OR vaccine service integration OR vaccine uptake OR Vaccine strategy OR vaccine delivery OR vaccine administration OR Immunization programme OR immunization program OR Immunisation programme OR Immunisation program OR Vaccination programme OR Vaccination program OR vaccine supply OR Vaccine distribution OR vaccine procurement OR vaccine promotion

**Resource name**	**Details of search**<br>**(Note: individual search strategies may vary depending on the resource**<br>**being used, record any variations here)**	**Number of hits**	**Details of key resources and actions taken**

Embase	#25) #24 AND #23<br>#24) #23 #22 #21 OR #20 OR #19 OR #18 OR #17 OR #16 OR #15 OR #14 OR 13 OR #12 OR #11 OR #10 OR #9 OR #8 OR #7<br>#23) #6 OR #5 OR #4 OR #3 OR #2 OR #1<br>#22 'vaccin* access'<br>#21 'vaccin* campaign*'<br>#20 'vaccin* rollout'<br>#19 'vaccin* implement*'<br>#18 'vaccin* integrat*'<br>#17 'vaccin* uptake'<br>#16 'vaccin* strateg*'<br>#15 'vaccin* delivery'<br>#14 'vaccin* administ*'<br>#13 'immuni? ation program*'<br>#12'vaccin* polic*'<br>#11 'vaccin* program*'<br>#10 'vaccin* supply'<br>#09 'vaccin* distribut*'<br>#08 'vaccin* procur*'<br>#07 'vaccin* promot*'<br>#6 'covid-19 virus vaccin*'<br>#5 'covid-19 immuni? ation'<br>#4 'sars-cov-2 immuni? ation'<br>#3 'sars-cov-2 vaccin*'<br>#2 'coronavirus disease 2019 vaccin*'<br>#1 'sars-cov-2 vaccine'/syn OR 'sars-cov-2 vaccine'	9,336<br>68, 082<br>54,692	Included two concepts.<br>1. Coronavirus Disease 2019 vaccine and its synonyms and MESH words from each database 2. Vaccine rollout or strategies and its synonyms<br>This narrowed down the search into papers on COVID-19 vaccine factors affecting vaccine delivery and uptake.<br><br>I did not exclude words that remove papers on individual factors of vaccine uptake for example vaccine hesitancy, vaccine intention, vaccine willingness.<br>Years included: 2020 to 2024.<br>Abstracts and titles
World Health Organization (WHO)	COVID–19 Vaccine campaign OR COVID-19 Vaccine promotion OR<br>COVID-19 Vaccine rollout OR COVID-19 Vaccine implementation OR COVID-19 vaccine service delivery OR	400	Searched COVID-19 vaccine campaign in the WHO website and it brought 400 papers incorporating the words showing in the second column. when the other words were put in the search tab, same results came out only shuffled with the results with the search word put Infront.

	COVID-19 vaccine uptake OR COVID-19 vaccine delivery OR COVID-19 Immunization programme OR COVID-19 Vaccine supply OR COVID-19 Vaccine access OR COVID-19 Vaccine policy<br>OR COVID-19 Vaccination program OR COVID-19 vaccine rollout OR<br>COVID-19 Vaccine distribution OR COVID-19 vaccine procurement OR COVID-19 Vaccine integration OR COVID19 Vaccine strategy OR COVID-19 vaccine administration.		
